# Digital device usage and psychological well-being in the post-COVID-19 era: insights from a cross-sectional survey

**DOI:** 10.1186/s40359-026-05205-x

**Published:** 2026-08-03

**Authors:** Md Jafrin Hossain, Munima Haque, Annajiat Alim Rasel, Md. Shohag Hossain, Mohammad Quamruzzaman, Umme Nusrat Jahan, Razoana Ayshee, Ariful Hasan Shuvo

**Affiliations:** 1 Knight Foundation School of Computing and Information Sciences, Florida International University, Miami, United States; 2 Department of Biotechnology, BRAC University, Dhaka, Bangladesh; 3 Department of Computer Science and Engineering, BRAC University, Dhaka, Bangladesh; 4 Department of Information and Communication Engineering, Daffodil International University, Dhaka, Bangladesh; 5 Department of Electrical and Electronic Engineering, World University of Bangladesh, Dhaka, Bangladesh; 6 Department of Computer Science and Engineering, East West University, Dhaka, Bangladesh; 7 Department of Economics, Shahjalal University of Science and Technology, Sylhet, Bangladesh

**Keywords:** COVID-19, Digital device usage, Psychological well-being, Health complaints, Online behavior, Cross-sectional study, Cluster analysis, Digital health

## Abstract

The COVID-19 pandemic accelerated digital technology adoption worldwide, yet limited evidence links post-pandemic multi-purpose online behavior with health outcomes and psychological well-being (PWB) in a single, integrated analysis. This study examines how digital device usage, breadth of online activity and self-reported Health Complaints relate to PWB across pre-COVID, during-COVID, and post-2023 periods. A cross-sectional survey of 622 respondents (collected in two pooled recruitment waves: Wave 1, $n{=}323$; Wave 2, $n{=}299$) assessed device usage across three temporal periods, online purpose diversity, Health Complaints (12 indicators), and PWB (Ryff’s 18-item scale). Spearman correlations with permutation-based *p*-values and Benjamini–Hochberg FDR correction were used. Group comparisons employed permutation tests with Cohen’s *d* effect sizes and bootstrap 95% confidence intervals. *K*-means clustering ($k{=}3$) identified behavioral segments. PWB was negatively associated with Health Complaints count (*ρ =-0.319*, $p_{\text {FDR}}<0.05$). Broader online purpose use correlated with more health effects (*ρ =0.202*, $p_{\text {FDR}}<0.05$). High online-purpose users (top quartile) reported significantly more Health Complaints than low users (*Δ =+1.55*, *d=0.54*, $p_{\text {FDR}}<0.001$). A partial Spearman correlation confirmed that this association persisted after controlling for total screen time ($\rho _{\text {partial}}{=}0.154$, $p_{\text {perm}}{<}0.001$, 95% CI [0.07, 0.23]), though the effect size was modest. Retrospective group-level estimates were higher for the during-COVID period than the pre-COVID baseline, with a lower estimate in Wave 2; these reflect between-wave, not within-person, comparisons. Three behavioral clusters emerged: low-usage/minimal-effects ($n{=}142$), high multi-device users ($n{=}291$), and higher health-effects/lower-PWB users ($n{=}189$). Among young, educated, urban adults, breadth of online activity was cross-sectionally associated with more self-reported adverse Health Complaints; both factors independently and collectively accounted for significant variance in PWB. These findings highlight the urgent need for digital wellness strategies that address activity diversity — not only total screen time — among young, educated, urban adults who represent the heaviest users of multi-purpose internet services in post-pandemic settings.

## Introduction

The COVID-19 pandemic fundamentally altered the relationship between individuals and digital technology. Lockdowns, remote work mandates, and social distancing measures accelerated the adoption of smartphones, computers, and internet-based services at an unprecedented pace [1–3]. While this digital transformation enabled continuity in education, employment, and social connection, emerging evidence suggests that increased screen time and diversified online activity may carry health costs, including sleep disruption, anxiety, eye strain, and reduced physical activity [4–6]. Large-scale reviews of pandemic-era digital media use further suggest that such behavioral shifts were accompanied by measurable declines in adolescent and young-adult mental health across multiple countries [7, 8].

Psychological well-being (PWB), as conceptualized by Ryff [9], encompasses six dimensions: self-acceptance, purpose in life, environmental mastery, personal growth, positive relations, and autonomy. Understanding how post-pandemic digital behaviors relate to these dimensions is critical for developing evidence-based wellness interventions. While several studies have examined early-pandemic technology use [1–3], few have captured retrospective usage estimates across three periods using a two-wave design [3]. Recent work has also extended this literature toward digital health literacy, app-based mental health support, and scalable digital interventions for student and community populations [10–14]. More recent contributions have also questioned whether large-scale technology adoption has the uniformly negative effects often assumed in public discourse, suggesting more nuanced, population-specific effects [15–17].

Theoretically, this work is situated within the *digital overload* framework, which posits that high volume and diversity of technology engagement can exceed individual cognitive and physiological coping capacity, producing adverse health consequences [6, 18]. Related constructs include the *displacement hypothesis*—whereby screen time crowds out health-promoting behaviors such as physical exercise and adequate sleep—and *technostress* models that link fragmented, multi-purpose device use to sustained arousal and attentional depletion [4, 5]. The eudaimonic model of PWB [9] provides the outcome framework, situating well-being not merely as the absence of distress but as the presence of purpose, mastery, and positive relations—dimensions that may be differentially eroded by digital overload. Behavioral health perspectives further suggest that the relationship between technology use and well-being is heterogeneous across individuals, motivating the segmentation approach employed here [19].

This study addresses three gaps in the literature. First, most COVID-era digital behavior studies focus on a single time point rather than comparing usage across multiple pandemic phases [1–3]. Second, limited evidence links the *breadth* of online activity—as opposed to total screen time—with health outcomes alongside PWB [19, 20]. Third, few analyses combine rigorous statistical testing with behavioral segmentation to identify distinct digital health profiles in South Asian populations, where internet-adoption trajectories and COVID-era behavioral shifts have been documented but under-integrated with psychological outcomes [19–21].

We analyze a normalized survey dataset of 622 respondents, examining: (1) how device and internet usage changed across three COVID periods; (2) the associations between online purpose diversity, Health Complaints, and PWB; (3) whether distinct user segments emerge that can inform targeted interventions. We employ permutation-based inference with false discovery rate (FDR) control and *K*-means clustering to provide a multi-faceted view of post-COVID digital well-being.

## Methods

### Study design and participants

Data were collected in two waves using a shared, harmonized survey instrument. Wave 1 ($n{=}323$) was disseminated primarily in 2022 with a supplementary round in 2025; Wave 2 ($n{=}299$) was disseminated in 2024. The two waves were pooled to increase analytic power and broaden student representation. The design is cross-sectional and not longitudinal: timestamp and demographic screening confirmed that no respondent appears in both waves, and the same individuals were not tracked over time. Because all participants reported usage retrospectively for the pre-COVID, during-COVID, and post-2023 periods, wave membership reflects only when a respondent was surveyed, not which pandemic period they experienced. Inclusion criteria required age *≥*18 years and regular internet access. The survey captured demographic information, device usage patterns across three retrospective periods (pre-COVID, during COVID, and post-2023), online activity purposes, self-reported Health Complaints, and psychological well-being. Similar cross-sectional digital mental health and well-being studies in student and young-adult populations have used related survey-based designs to characterize technology use, symptom burden, and service engagement patterns [22–24].

### Measures

#### Device and internet usage

Respondents rated their usage of phone, internet, and computer across three periods (pre-COVID, during COVID, post-2023) on an 8-point ordinal scale anchored at 1 (*never*/less than 30 minutes per day) and 8 (*more than 10 hours per day*), with intermediate points representing approximately equal intervals of daily use time (2 = 30–60 min/day; 3 = 1–2 hrs/day; 4 = 2–3 hrs/day; 5 = 3–5 hrs/day; 6 = 5–7 hrs/day; 7 = 7–10 hrs/day). The scale is ordinal rather than interval, which was accounted for by the use of Spearman rank correlations throughout. Change indicators captured perceived shifts in usage during COVID. As no established validated device-use instrument was employed (e.g., the Smartphone Addiction Scale–Short Version [25]), the scale constitutes a study-specific measure and direct comparisons with externally validated tools are not possible. Subsequent validation studies across multiple cultural settings have continued to support the utility of short smartphone-addiction screening instruments for young people and student populations [26–32].

#### Online purpose diversity

Nine binary items assessed whether respondents used the internet for educational, messaging, video, gaming, social media, shopping, news, official work, and other purposes. The sum yielded an *online purposes count* (range 0–9).

#### Health complaints

Twelve binary indicators measured self-reported health symptom awareness: self-reported anxiety-related symptoms, insomnia, depressive symptoms, concentration breaks, headache, shortness of temper, sleep disorder, face-to-face discomfort, decreased attention, hearing loss, eyesight problems, and eye discomfort. A *Health Complaints count* was computed (range 0–12). This checklist was developed specifically for this study and has not undergone formal psychometric validation. Although the health effect indicators were not derived from a previously validated scale, the items were constructed based on commonly reported digital health symptoms in prior literature [4, 6]. This approach is appropriate for exploratory studies where standardized instruments may not fully capture context-specific behavioral patterns. Findings based on these items should therefore be interpreted as prevalence estimates of self-reported symptom awareness rather than clinically diagnosed conditions; established instruments such as the PHQ-9 [33], GAD-7 [34], and Pittsburgh Sleep Quality Index [35] would provide greater diagnostic precision and cross-study comparability.

#### Psychological well-being

PWB was measured using an adapted 18-item Ryff scale [9, 36] scored on a 1–7 Likert format across six dimensions: self-acceptance (3 items), purpose in life (2 items), environmental mastery (3 items), personal growth (4 items), positive relations (3 items), and autonomy (3 items). Dimension means and an overall PWB total were computed.

The internal consistency of the adapted scale was assessed using Cronbach’s *α*, computed on properly reverse-scored items (negatively-worded items recoded as *8 - x* prior to analysis). The overall 18-item PWB composite demonstrated acceptable internal consistency ($\alpha {=}0.727$). Subscale-level alphas were substantially lower due to short subscale length and the adapted translation: self-acceptance ($\alpha {=}0.624$), purpose in life ($\alpha {=}0.228$), environmental mastery ($\alpha {=}0.296$), personal growth ($\alpha {=}0.396$), positive relations ($\alpha {=}0.393$), and autonomy ($\alpha {=}0.357$). All subscale alphas fall below the conventional 0.70 threshold. Given these reliability limitations, all PWB-related inferences in this study are conducted at the composite total-score level only; subscale-level comparisons should not be interpreted.

### Statistical analysis

All analyses were conducted in Python using NumPy, Pandas, and SciPy. Spearman rank correlations assessed monotonic associations between key continuous variables. Statistical significance was evaluated using permutation tests (2 000 permutations) rather than parametric assumptions, given the ordinal nature of several variables. Multiple comparison correction was applied via the Benjamini–Hochberg (BH) false discovery rate procedure at $\alpha {=}0.05$.

Group comparisons between low (Q1) and high (Q3) online purpose users employed permutation tests for difference in means (2 000 permutations). Effect sizes were quantified using Cohen’s *d* with 95% bootstrap confidence intervals (2 000 resamples). *K*-means clustering was applied to standardized features combining key behavioral and health variables with period-specific usage. The number of clusters was evaluated by computing the mean silhouette coefficient for $k{=}2$ through $k{=}5$ (12 random initializations per *k*, seed fixed for reproducibility). Silhouette scores were: $k{=}2$, $\bar{s}{=}0.136$; $k{=}3$, $\bar{s}{=}0.111$; $k{=}4$, $\bar{s}{=}0.121$; $k{=}5$, $\bar{s}{=}0.094$. Although $k{=}2$ yielded the highest silhouette coefficient, $k{=}3$ was selected because it produced three theoretically distinct and interpretable behavioral profiles (low-usage/minimal-effects; high multi-device; high health-effects/lower-PWB) that better aligned with the study’s conceptual framework. All silhouette scores were low ($\bar{s}{<}0.14$), reflecting overlapping clusters typical of survey-based behavioral data. PCA projection onto two dimensions facilitated visualization. In behavioral survey data, such overlap is expected due to the continuous and non-discrete nature of human activity patterns. Therefore, cluster selection was guided not only by separation metrics but also by interpretability and theoretical relevance. All analyses are descriptive and associative in nature; no causal or temporal inferences can be drawn from the observed relationships. All analyses conducted in this study are descriptive and associative in nature. Given the cross-sectional design, no causal or temporal inferences can be drawn from the observed relationships. The decomposition of associations is intended for exploratory purposes only and does not imply a causal decomposition process.

### Missing data

Post-2023 device usage variables had approximately 41% missingness, attributable to Wave 1 not receiving these items by design (wave-specific, structural missingness). Data were assumed missing at random (MAR) on the basis that non-receipt was determined entirely by wave assignment rather than by the values of the missing variables themselves. Age had 6.4% missingness. Median imputation was applied exclusively for the *k*-means clustering procedure to enable complete-case matrix operations; all other analyses were conducted on available cases without imputation. A sensitivity comparison of imputed versus complete-case clustering results was not conducted, which represents a limitation of the clustering analysis.

## Results

### Demographic characteristics

**Fig. 1:**
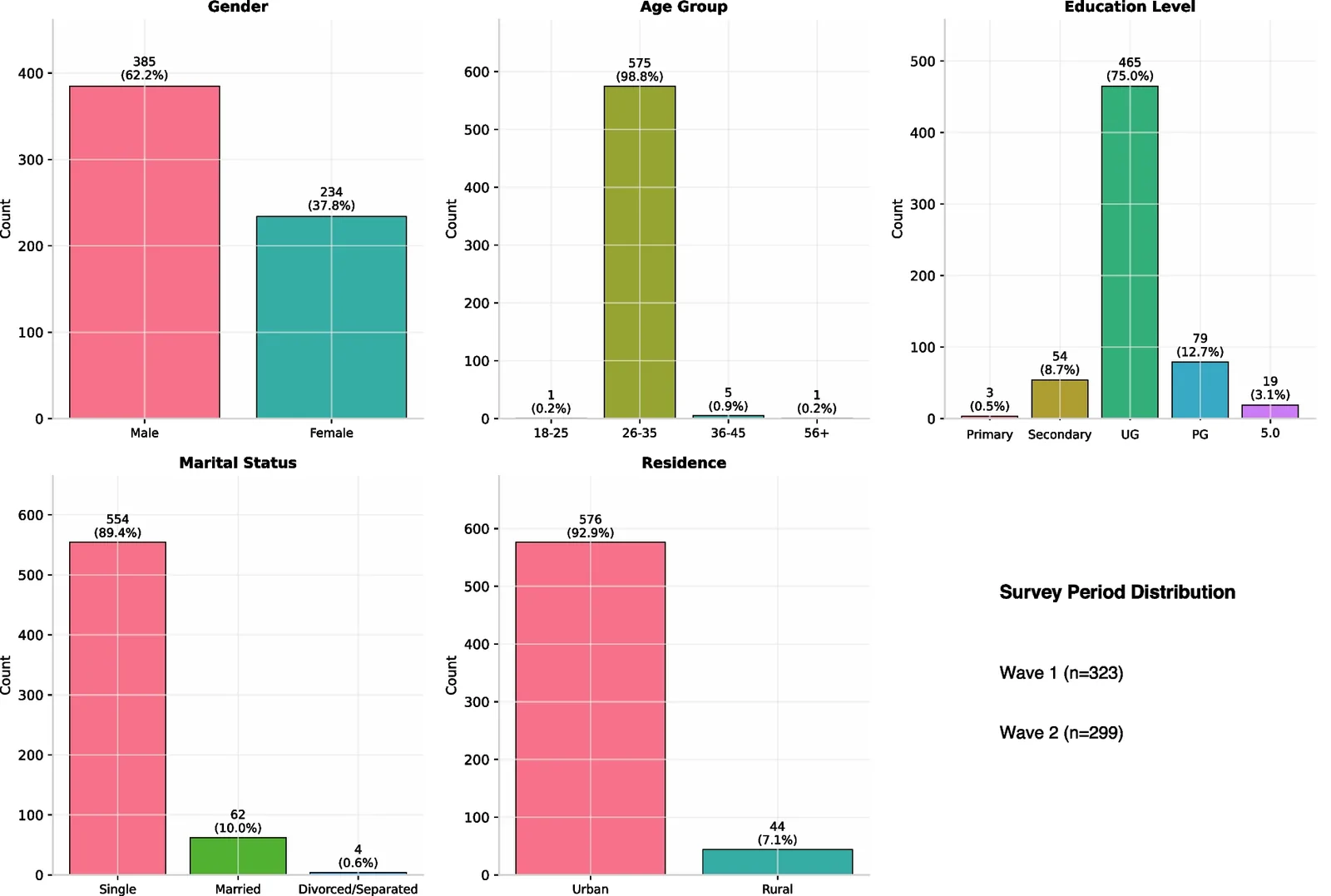
Demographic characteristics of the study sample ($N{=}622$). Six-panel bar chart showing the distribution of gender, age, education, marital status, and residence

The sample comprised 622 respondents (385 male [61.9%], 234 female [37.6%]). The modal age group was 26–35 years (98.8%). The majority held undergraduate (74.8%) or postgraduate degrees (12.7%). Most respondents were single (89.1%) and resided in urban areas (92.6%). Table 1 presents the full demographic breakdown, and Fig. 1 visualizes the distributions.

**Table 1:** Demographic characteristics of the study sample ($N{=}622$)

Variable	Category	*n*	%
Gender	Male	385	61.9
	Female	234	37.6
Age	18–25	1	0.2
	26–35	575	98.8
	36–45	5	0.9
	56+	1	0.2
Education	Primary	3	0.5
	Secondary	54	8.7
	Undergraduate	465	74.8
	Postgraduate	79	12.7
	Other	19	3.1
Marital Status	Single	554	89.1
	Married	62	10.0
	Divorced/Separated	4	0.6
Residence	Urban	576	92.6
	Rural	44	7.1

Missing data excluded from percentages: Gender ($n{=}3$), Age ($n{=}40$), Education ($n{=}2$), Marital Status ($n{=}2$), Residence ($n{=}2$)

#### Comparability of the two waves

The two waves were compared on key characteristics. They differed in gender composition (Wave 1: 54.2% male; Wave 2: 70.9% male; *p<0.001*), education (*p<0.001*), marital status (*p<0.001*), and profession (*p<0.001*), but did not differ in residence ($p{=}0.08$) or in psychological well-being (Wave 1 $M{=}4.81$; Wave 2 $M{=}4.72$; $p{=}0.16$). These differences are consistent with the two waves drawing on partly different recruitment pools. To ensure they did not drive the findings, the multivariate model was re-estimated with a wave indicator (Multivariate predictors of psychological well-being section).

### Device usage across COVID periods

**Fig. 2:**
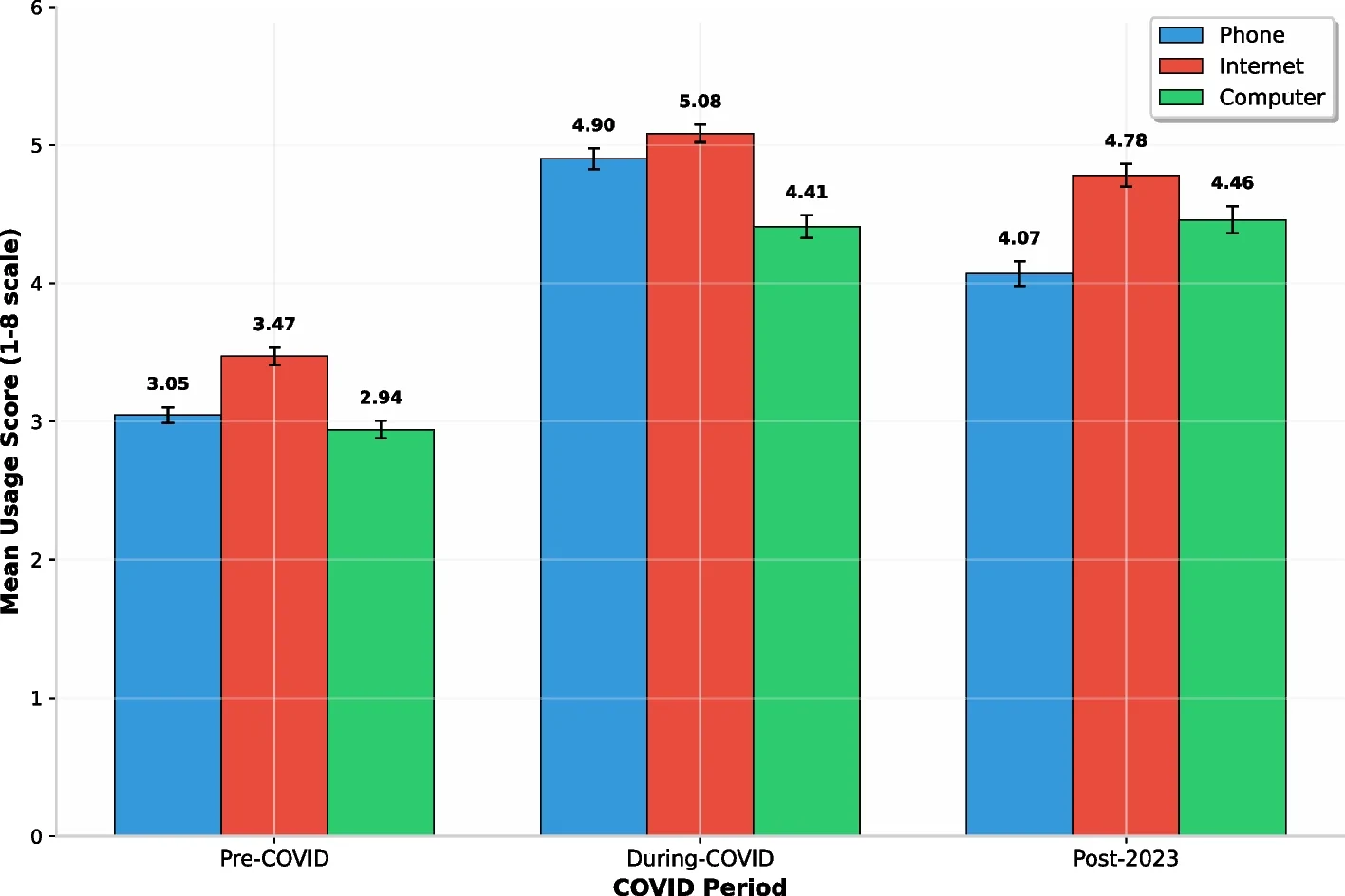
Device usage across COVID periods. All three device types show a marked increase during COVID followed by partial post-2023 decline, with internet usage remaining most elevated post-pandemic. Mean usage on a 1–8 ordinal scale; error bars represent standard errors

**Fig. 3:**
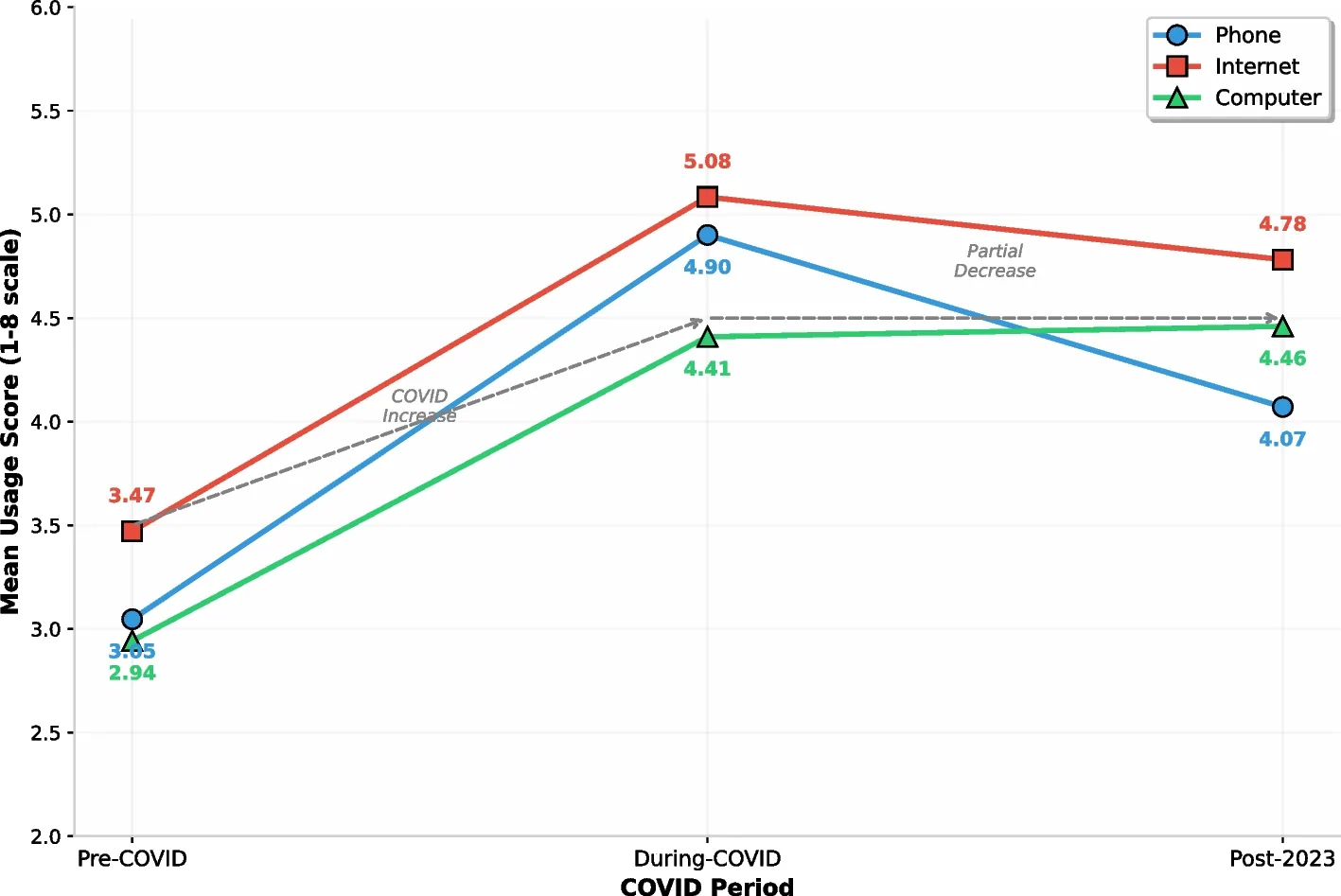
Device usage trajectory across COVID periods. Line plot showing cross-wave differences in retrospectively reported device usage across three periods. Note: values reflect group-level means, not individual-level longitudinal tracking for phone, internet, and computer usage

Comparing mean usage across retrospective periods and waves, scores were higher during COVID relative to pre-COVID baselines, with some reduction observed in Wave 2 (Figs. 2 and 3). Mean phone usage rose from 3.05 (pre-COVID) to 4.90 (during COVID), then partially receded to 4.07 (post-2023). Internet usage followed a similar trajectory ($3.47 \rightarrow 5.08 \rightarrow 4.78$). Computer usage increased from 2.94 to 4.41 and remained stable at 4.46 post-2023. Figure 3 presents the cross-wave usage trajectory across the three retrospective periods, illustrating the lasting pandemic footprint in device engagement. These group-level estimates are consistent with a lasting pandemic footprint, though between-wave differences should not be interpreted as within-person change compared to pre-COVID baselines. Table 2 summarizes these values.

**Table 2:** Mean device usage by COVID period (1–8 Scale)

Device	Pre-COVID		During COVID		Post-2023	
	*M*	*SD*	*M*	*SD*	*M*	*SD*
Phone	3.05	1.42	4.90	1.86	4.07	1.68
Internet	3.47	1.58	5.08	1.59	4.78	1.56
Computer	2.94	1.54	4.41	2.04	4.46	1.87

Post-2023 data available for$n{=}299$(Wave 2 only)

### Online activity and health complaints prevalence

**Fig. 4:**
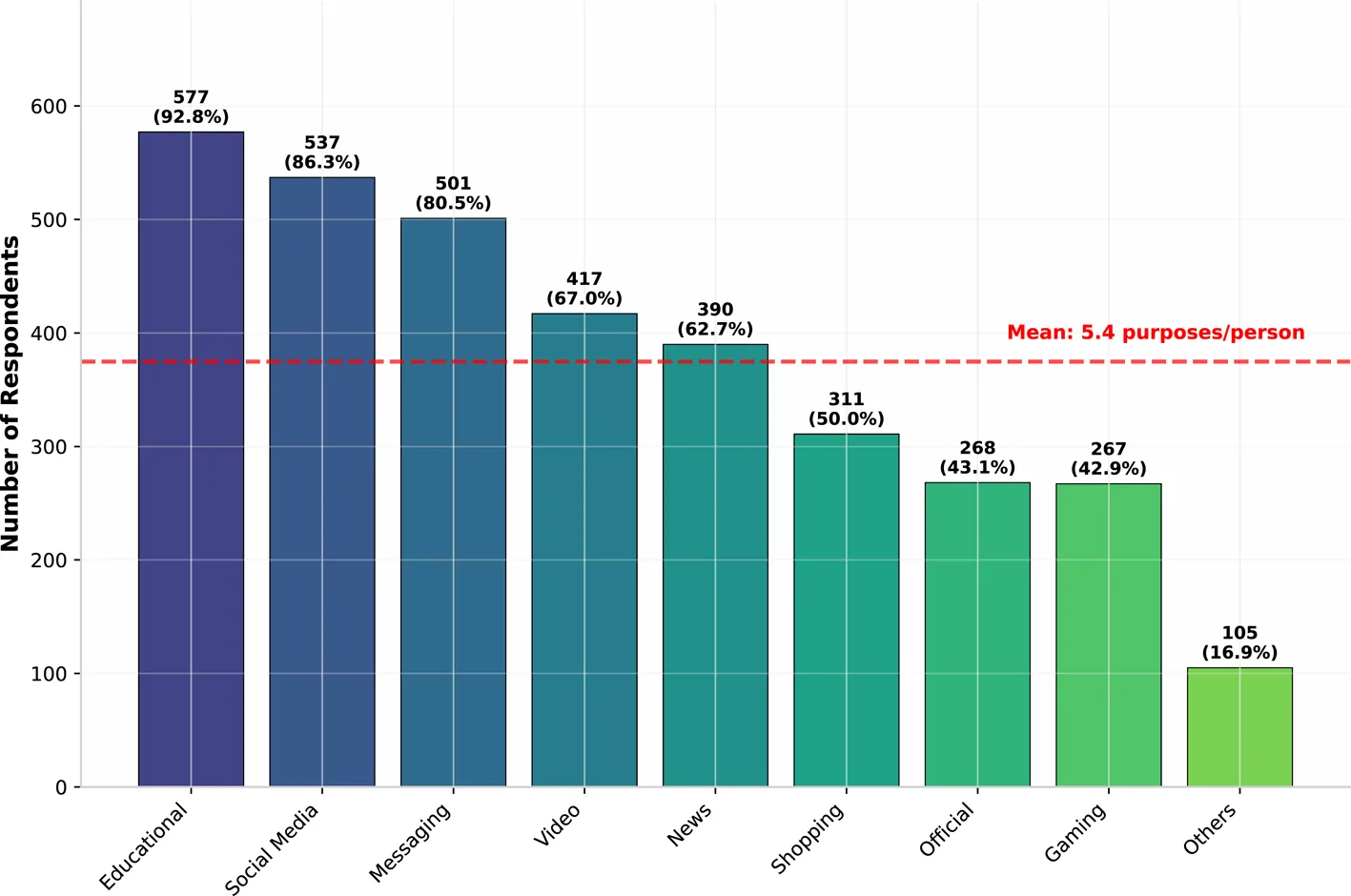
Online activity purposes ($N{=}622$). Vertical bar chart showing the percentage of respondents engaging in each of nine internet activity categories. Educational use (92.8%) and social media (86.3%) were the most common

**Fig. 5:**
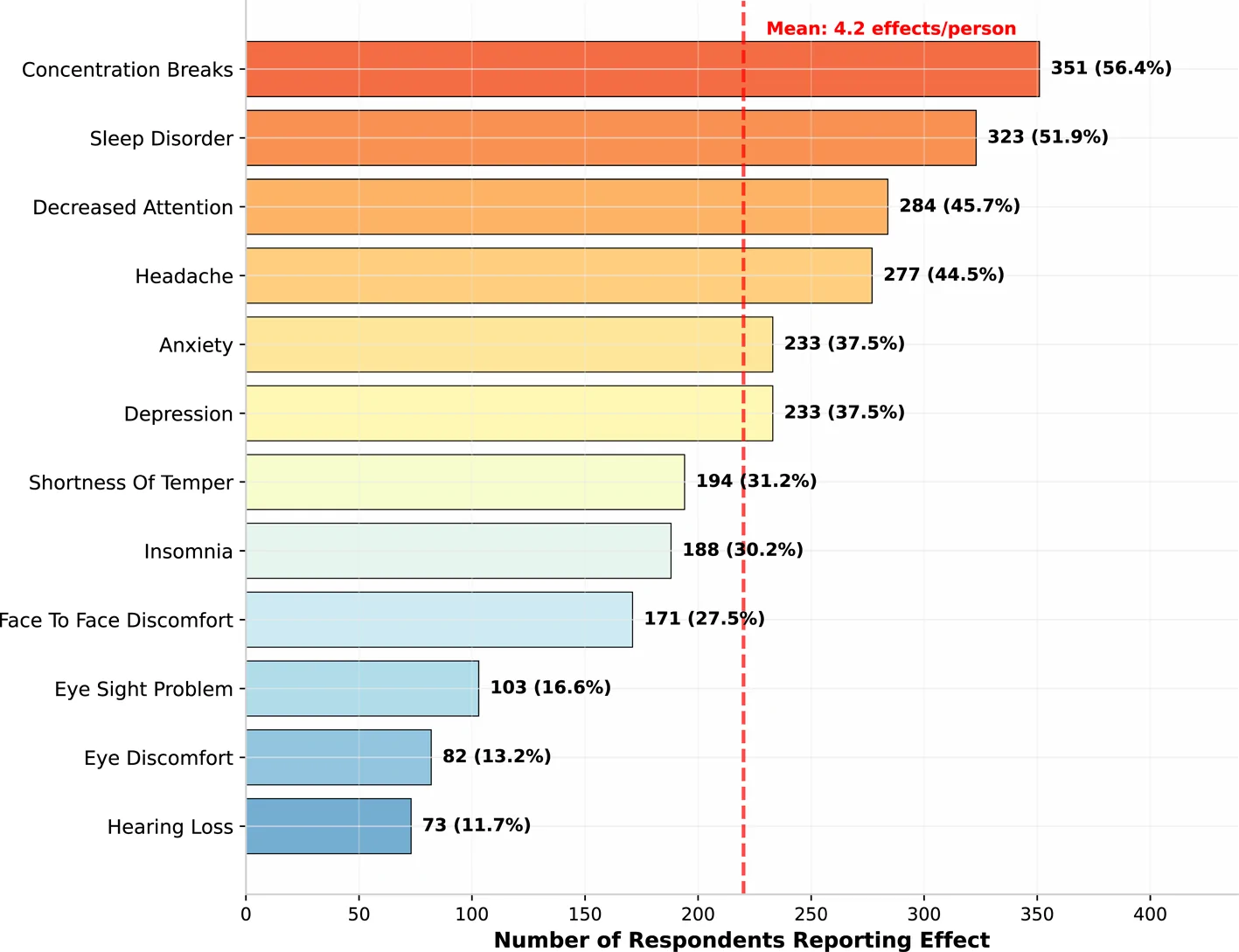
Prevalence of self-reported Health Complaints ($N{=}622$). Over half the sample reported concentration breaks (56.4%) and sleep disorder (51.9%); decreased attention (45.7%) and headache (44.5%) were also highly prevalent, suggesting a broad cognitive and somatic symptom burden

Respondents engaged in an average of 5.42 distinct online purposes (*SD*=2.06). Educational use (92.8%), social media (86.3%), and messaging (80.5%) were the most prevalent activities (Fig. 4). The mean number of reported Health Complaints was 4.25 (*SD*=2.93), with concentration breaks (56.4%), sleep disorder (51.9%), and decreased attention (45.7%) among the most commonly reported complaints (Fig. 5).

### Correlations among key variables

**Fig. 6:**
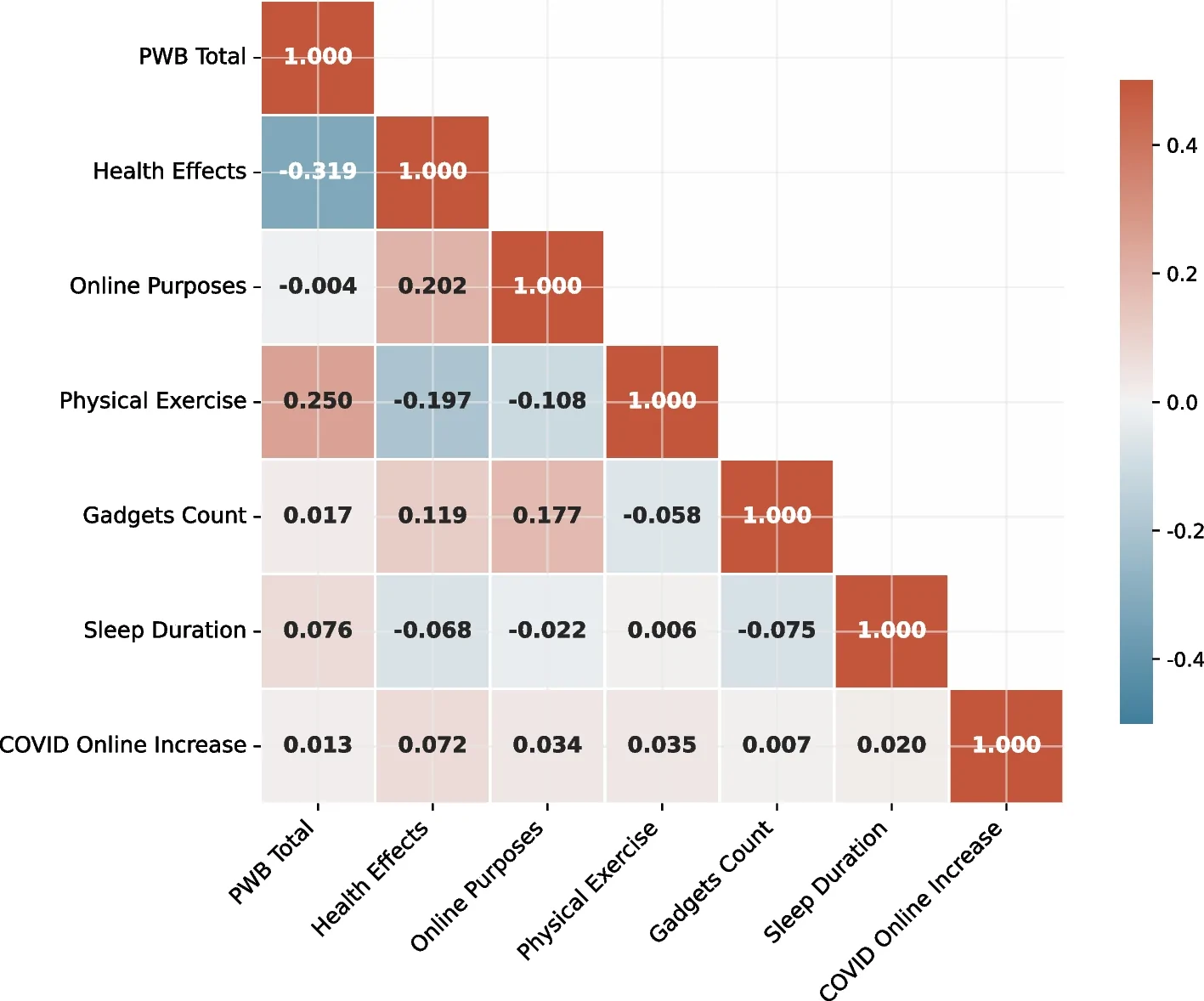
Spearman rank correlation matrix among key study variables. The PWB–Health Complaints association ($\rho {=}{-}0.319$) is the strongest in the dataset; physical exercise shows protective associations with both PWB and Health Complaints. Significant correlations ($p_{\text {FDR}}<0.05$) annotated with asterisks; color intensity represents magnitude

Spearman rank correlations with permutation-based *p*-values and BH-FDR correction are presented in Table 3. The strongest association was between PWB total and Health Complaints count ($\rho {=}{-}0.319$, $p_{\text {FDR}}<0.05$), indicating that respondents reporting more health complaints had lower psychological well-being. Online purpose count was positively associated with Health Complaints ($\rho {=}0.202$, $p_{\text {FDR}}<0.05$), suggesting that broader online engagement corresponds to more Health Complaints. Physical exercise was inversely related to Health Complaints ($\rho {=}{-}0.197$, $p_{\text {FDR}}<0.05$) and positively associated with PWB ($\rho {=}0.250$, $p_{\text {FDR}}<0.05$), consistent with the broader exercise and mental health literature [37, 38]. Figure 6 displays the full correlation matrix. To test whether breadth of activity relates to Health Complaints independently of usage volume, a partial Spearman correlation was computed between online-purpose count and health-complaints count controlling for total screen time (sum of phone, internet, and computer usage during COVID). The association remained positive but modest ($\rho _{\text {partial}}{=}0.154$, $p_{\text {perm}}{<}0.001$, 95% CI [0.07, 0.23]; raw $\rho {=}0.20$; $n{=}621$), indicating a small but statistically significant independent contribution of activity diversity to symptom burden beyond total screen time alone. The modest magnitude of this effect ($\rho _{\text {partial}}{=}0.154$) warrants cautious interpretation: while the association is robust to confounding by overall usage volume, it accounts for only a small proportion of variance in Health Complaints.

**Table 3:** Spearman rank correlations among key variables (FDR-corrected)

Variable A	Variable B	*ρ*	$p_{\text {perm}}$	$p_{\text {FDR}}$
PWB Total	Health Complaints Count	−0.319	0.0005	0.0105
PWB Total	Physical Exercise	0.250	0.0005	0.0053
Health Complaints Count	Online Purposes Count	0.202	0.0005	0.0035
Health Complaints Count	Physical Exercise	−0.197	0.0005	0.0026
Online Purposes Count	Gadgets Count	0.177	0.0005	0.0021
Health Complaints Count	Gadgets Count	0.119	0.0025	0.0088
Online Purposes Count	Physical Exercise	−0.108	0.0070	0.0210
PWB Total	Sleep Duration	0.076	0.0590	0.1549
Gadgets Count	Sleep Duration	−0.075	0.0590	0.1377
Health Complaints Count	COVID Online Increase	0.072	0.0720	0.1512

*$p_{\text {FDR}} < 0.05$. Permutation tests with 2 000 iterations. FDR correction via Benjamini–Hochberg procedure. Only the top 10 correlations (by *|ρ |*) are shown

### PWB and health complaints by survey period

**Fig. 7:**
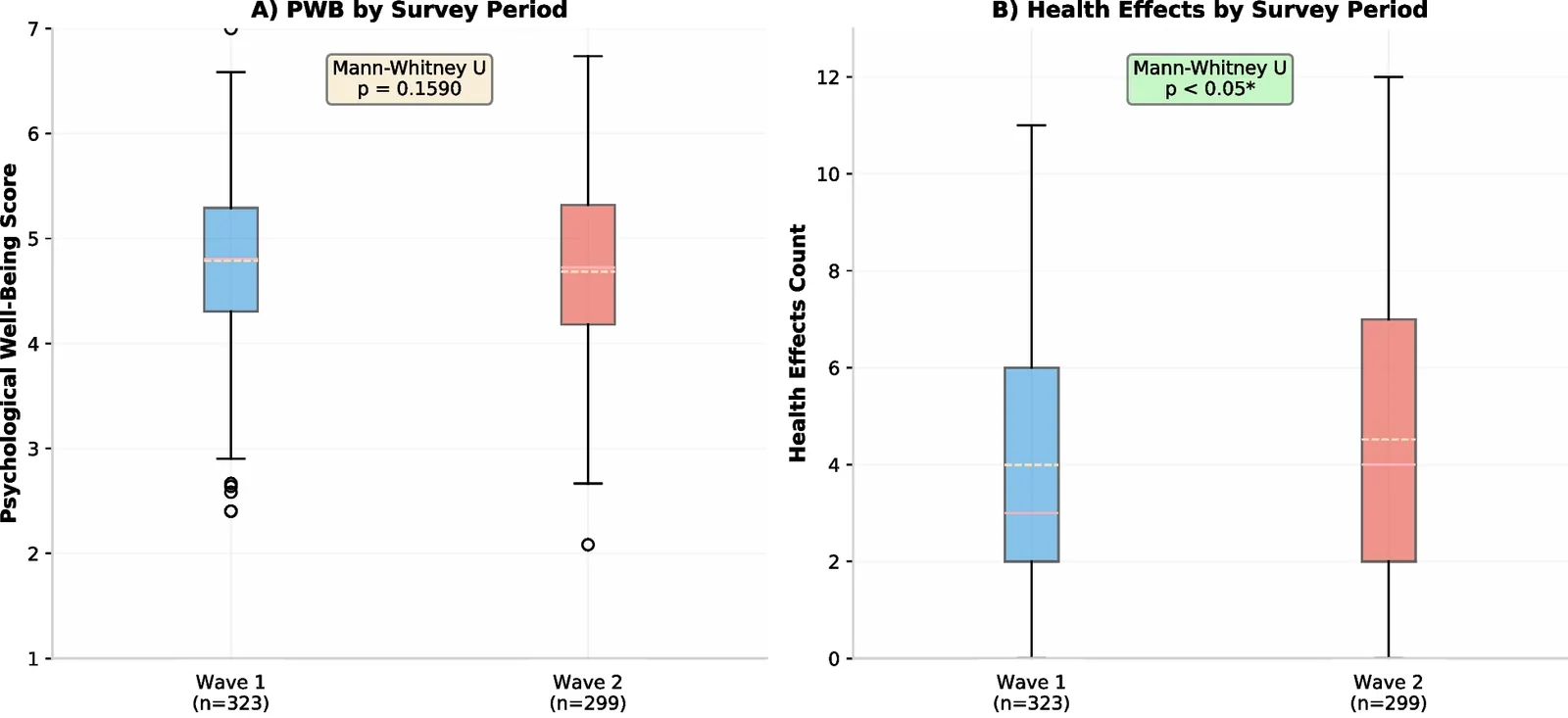
Psychological well-being and Health Complaints by survey period. **A** Psychological well-being (PWB) box plot and **B** Health Complaints box plot. Box plots comparing distributions between Wave 1 ($n{=}323$) and Wave 2 ($n{=}299$). Health Complaints count was significantly higher in Wave 2 ($p{=}0.018$)

**Fig. 8:**
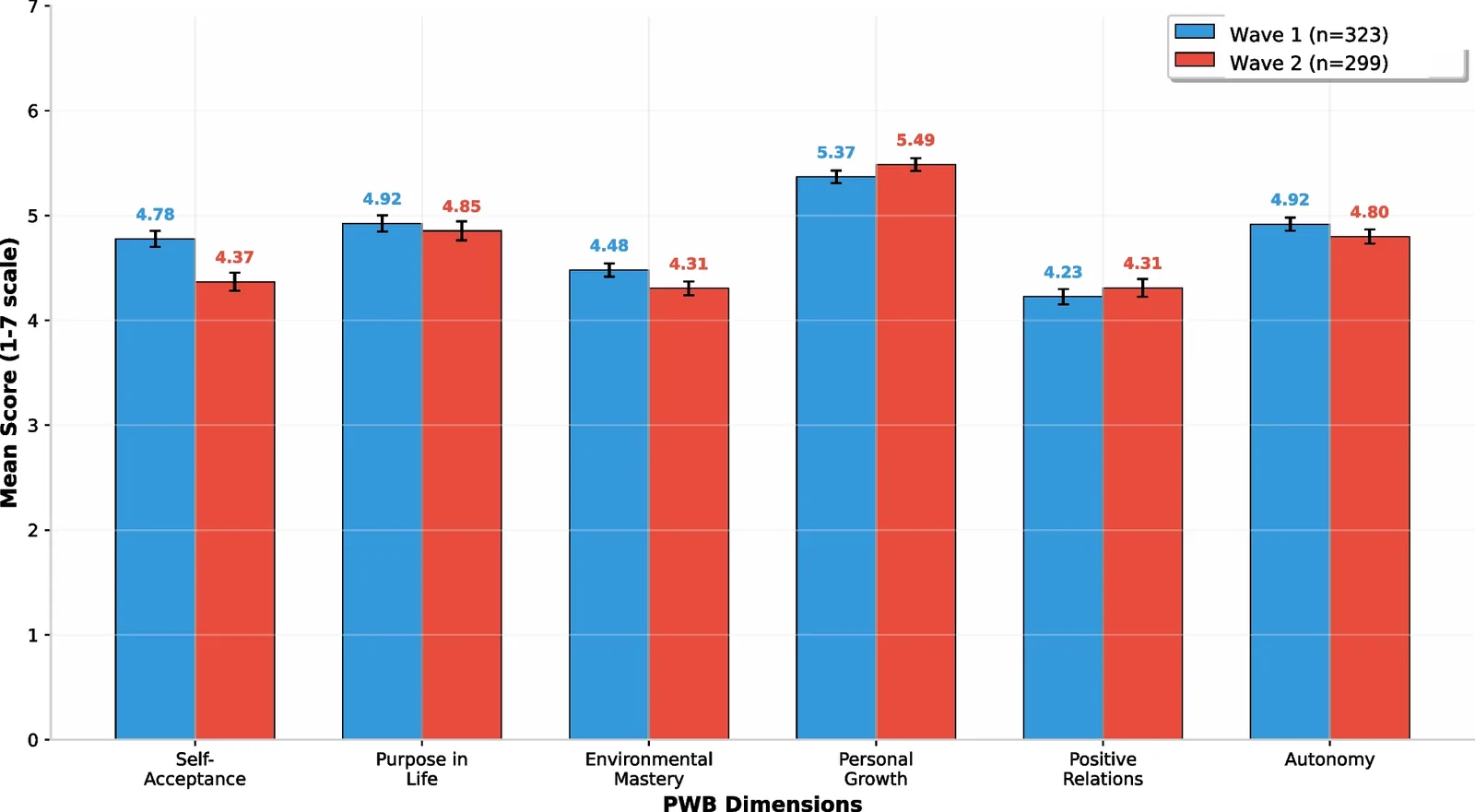
PWB dimension scores by data-collection wave. Grouped bar chart showing mean scores on each of six Ryff PWB subscales for Wave 1 and Wave 2. Error bars represent standard errors

Figure 7 compares PWB and Health Complaints distributions by survey period. Mean PWB was 4.79 (*SD*=0.78) in Wave 1 and 4.69 (*SD*=0.82) in Wave 2 (Mann–Whitney U=51,104, *p*=0.159). Mean Health Complaints count was 3.99 (*SD*=2.88) versus 4.52 (*SD*=2.96; U=43,015, *p*=0.018). Figure 8 provides a breakdown of PWB dimension scores by period. Because the two waves differ demographically (Demographic characteristics section), this between-wave difference may partly reflect sample composition rather than a temporal effect; consistent with this, wave was not a significant predictor of well-being once demographics were controlled (Multivariate predictors of psychological well-being section).

### Low vs. high online purpose users

**Fig. 9:**
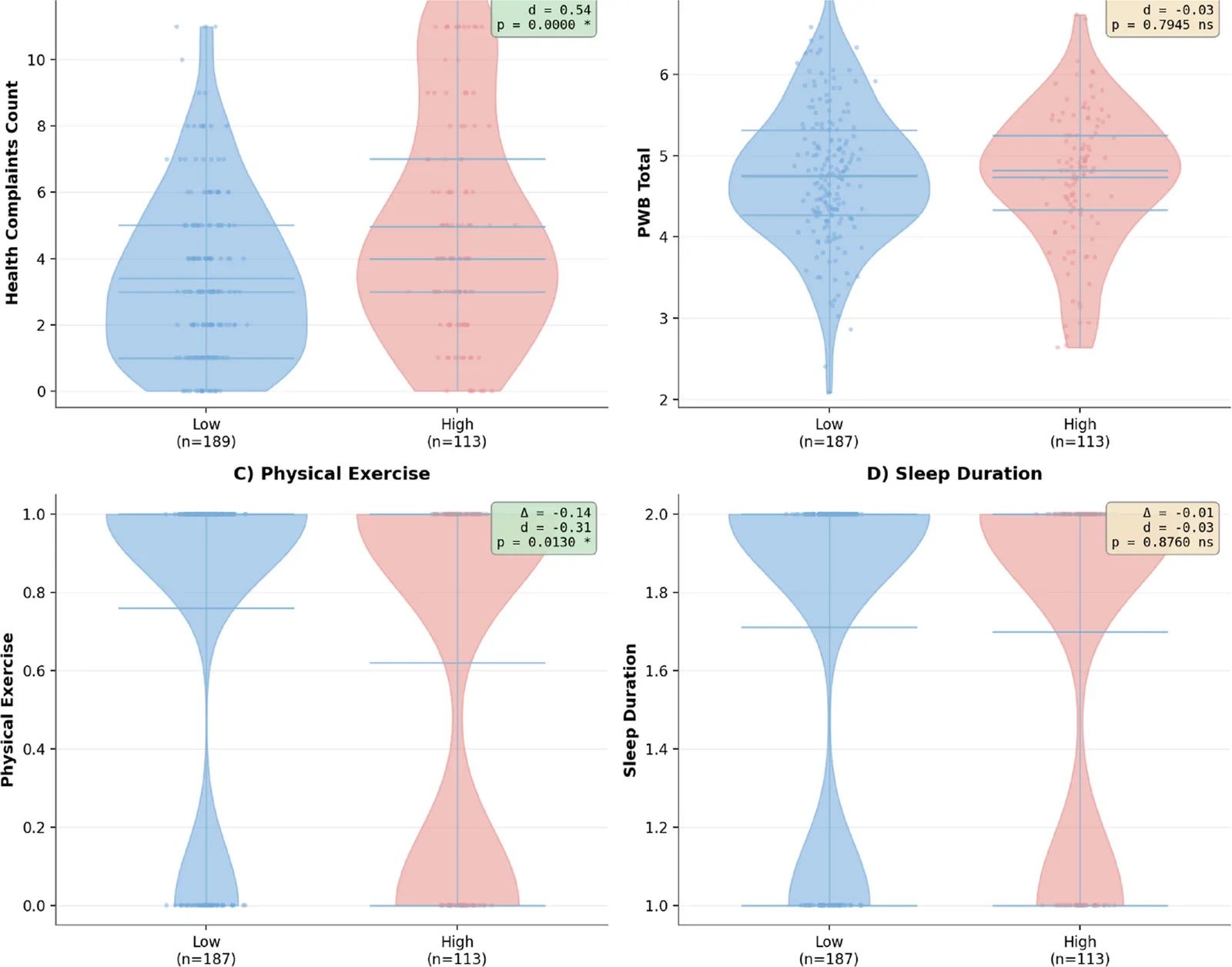
Comparison of low vs. high online purpose users. Violin plots showing distributions of Health Complaints count, PWB total, physical exercise, and sleep duration for Q1 (*≤*4 purposes) and Q3 (*≥*7 purposes) groups. Significant differences marked with asterisks

Respondents were stratified into low (Q1, *≤*4 purposes; $n{=}189$) and high (Q3, *≥*7 purposes; $n{=}113$) online activity groups. Table 4 presents group comparisons. High online-purpose users reported significantly more Health Complaints ($\Delta {=}+1.55$, $d{=}0.54$, 95% CI [0.30, 0.79], $p_{\text {FDR}}<0.001$), representing a moderate effect size. Physical exercise was modestly lower among high-purpose users ($d{=}-0.31$, $p_{\text {FDR}}{=}0.023$). PWB and sleep duration differences were not statistically significant after FDR correction (Fig. 9).

**Table 4:** Comparison of low vs. high online purpose users (Q1 vs. Q3)

Variable	Low *M* (*SD*)	High *M* (*SD*)	*Δ*	*d*	95% CI	$p_{\text {FDR}}$
Health Complaints Count	3.41 (2.49)	4.96 (3.36)	+1.55	0.54	[0.30, 0.79]	<0.001*
PWB Total	4.76 (0.82)	4.73 (0.83)	−0.03	−0.03	[−0.27, 0.21]	1.000
Physical Exercise	0.76 (0.43)	0.62 (0.49)	−0.14	−0.31	[−0.54, −0.06]	0.023*
Sleep Duration	1.71 (0.45)	1.70 (0.46)	−0.01	−0.03	[−0.27, 0.21]	0.894

*$p_{\text {FDR}} < 0.05$. Low = Q1 (*≤*4 purposes, $n{=}189$); High = Q3 (*≥*7 purposes, $n{=}113$). *d* = Cohen’s *d*; CI = bootstrap 95% confidence interval (2 000 resamples)

### Behavioral segmentation via *K*-means clustering

**Fig. 10:**
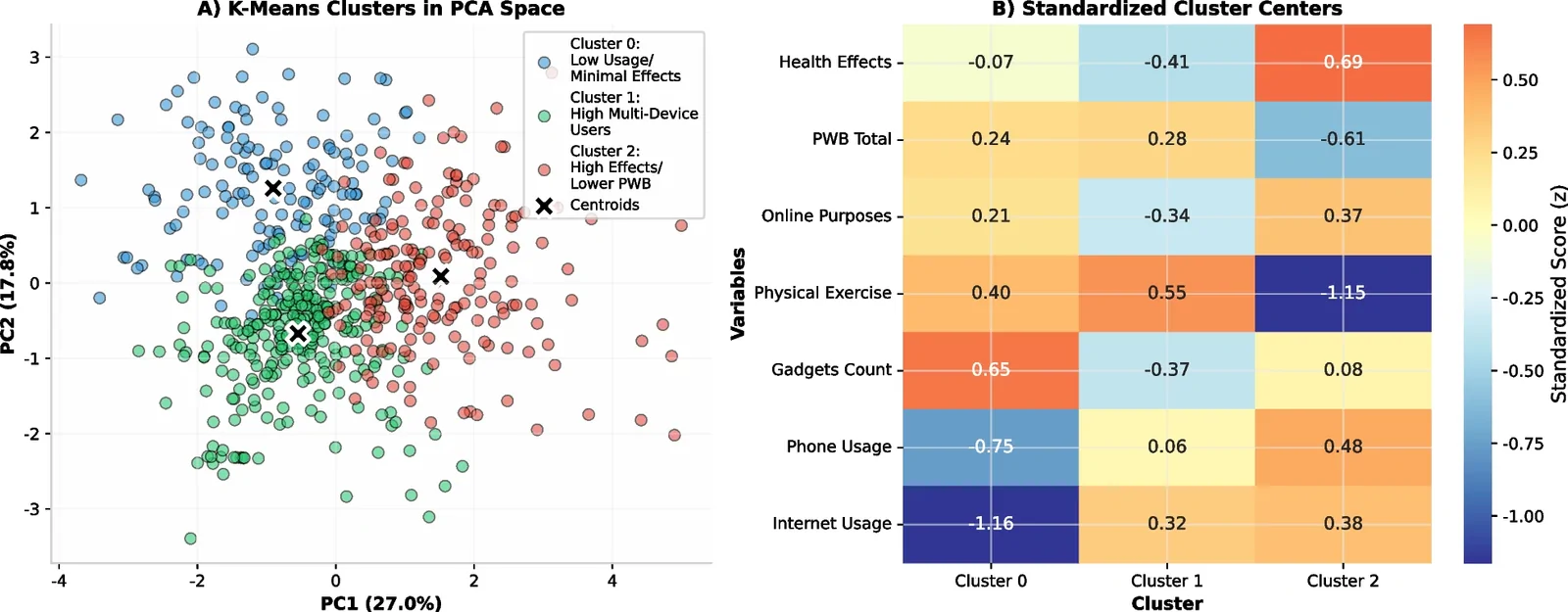
*K*-means clustering results ($k{=}3$) reveal three behaviorally distinct segments. **A** PCA projection of the three identified clusters. **B** Heatmap of standardized cluster centers across the study variables. Cluster 2 (30.4% of the sample) represents the high-risk profile, characterized by elevated Health Complaints and below-average PWB, whereas Cluster 1 (46.8%) represents high multi-device use with relatively neutral health

*K*-means clustering ($k{=}3$) on standardized features identified three distinct segments (Fig. 10). Cluster 0 ($n{=}142$, 22.8%) comprised low-usage individuals with minimal Health Complaints and baseline PWB. Cluster 1 ($n{=}291$, 46.8%) captured high multi-device/internet users with diverse online purposes and relatively neutral Health Complaints. Cluster 2 ($n{=}189$, 30.4%) exhibited higher reported Health Complaints, lower PWB and physical exercise, with moderate usage levels. Table 5 presents standardized cluster centers for key variables.

**Table 5:** Standardized *K*-means cluster centers for key variables ($k{=}3$)

Variable	Cluster 0	Cluster 1	Cluster 2
	($n{=}142$, 22.8%)	($n{=}291$, 46.8%)	($n{=}189$, 30.4%)
Health Complaints Count	−0.07	−0.41	0.69
PWB Total	0.24	0.28	−0.61
Online Purposes Count	0.21	−0.34	0.37
Physical Exercise	0.40	0.55	−1.15
Gadgets Count	0.65	−0.37	0.08
Phone Usage	−0.75	0.06	0.48
Internet Usage	−1.16	0.32	0.38

Values are *z*-scored (mean *= 0*,$SD{=}1$). Cluster 0 = Low-usage/minimal-effects group; Cluster 1 = High multi-device users with neutral health; Cluster 2 = High Health Complaints/lower PWB (risk group). *K*-means with 12 random initializations; features standardized prior to clustering.$k{=}3$selected on theoretical interpretability grounds (mean silhouette$\bar{s}{=}0.111$;$k{=}2$had highest silhouette$\bar{s}{=}0.136$but yielded only two profiles)

### Analysis of indirect association patterns between online activity and well-being

To characterize how the observed cross-sectional associations partition statistically, we computed a descriptive decomposition of the online-purposes–health-effects–PWB relationship using bootstrap resampling ($n_{\text {boot}}{=}2{,}000$, seed *=42*; $n{=}620$). We emphasize that this is not a test of causal decomposition: with all three variables measured at a single time point, temporal precedence cannot be established, and cross-sectional indirect association component estimates are known to be biased relative to their longitudinal counterparts [39, 40]. The results below are therefore reported solely as a descriptive summary of how the bivariate associations partition, not as evidence of an underlying causal mechanism.

The association between online purposes and Health Complaints was positive (standardized $a{=}0.331$), and the association between Health Complaints and PWB, adjusting for online purposes, was negative ($b{=}{-}0.095$). The product term was $ab{=}{-}0.031$ (bootstrap 95% CI [*-0.044*, *-0.019*]), while the adjusted direct association ($c'{=}0.019$) and the total bivariate association ($c{=}{-}0.013$) were both near zero. This pattern is consistent with the interpretation that the cross-sectional link between online activity breadth and PWB is dominated by a statistical overlap with self-reported Health Complaints, but the direction of any underlying process cannot be inferred from these data. Prospective longitudinal or ecological-momentary-assessment designs are required before any statistical association pattern can be tested [39, 41].

### Multivariate predictors of psychological well-being

Predictors were selected on theoretical grounds rather than by data-driven search: health-complaints count operationalizes the proximal symptom burden central to the digital-overload framework; online-purpose count is the primary breadth-of-activity exposure; education indexes socioeconomic resources, a well-established correlate of eudaimonic well-being; and age and gender are standard demographic controls in well-being research.

To address potential confounding by demographic variables, an ordinary least squares (OLS) regression was estimated with PWB total as the dependent variable and five predictors: Health Complaints count, online purposes count, age, gender, and education ($n{=}578$ complete cases). Results are presented in Table 6.

The model was statistically significant ($F(5, 572){=}18.73$, $p{<}0.001$) and explained 13.3% of variance in PWB (adjusted $R^2{=}0.133$). Health Complaints count remained the strongest predictor after controlling for demographic variables ($B{=}{-}0.091$, $SE{=}0.011$, $p{<}0.001$; 95% CI [*-0.113*, *-0.069*]): each additional health symptom was associated with a 0.091-unit decrease in composite PWB. Education was a significant positive predictor ($B{=}0.251$, $SE{=}0.065$, $p{<}0.001$; 95% CI [0.124, 0.378]), indicating that higher educational attainment was associated with better well-being independently of digital behavior. Online purpose count ($p{=}0.096$), age ($p{=}0.080$), and gender ($p{=}0.253$) did not reach conventional significance thresholds, consistent with the decomposition analysis indicating that online activity breadth overlaps with PWB primarily through its co-occurrence with Health Complaints rather than through a direct association. As a sensitivity analysis, the model was re-estimated with a wave indicator added. The health-complaints–PWB coefficient was essentially unchanged (*B≈ -0.09*, *p<0.001*) and wave itself was non-significant ($p{=}0.43$), indicating the central association is robust to between-wave compositional differences. Full regression coefficients, standard errors, and confidence intervals are reported in Table 6.

**Table 6:** OLS regression: predictors of psychological well-being ($N{=}578$)

Predictor	*B*	*SE*	*t*	*p*	95% CI
(Intercept)	5.027	0.418	12.031	<0.001*	[4.206, 5.848]
Health Complaints Count	−0.091	0.011	−8.003	<0.001*	[*-0.113*, *-0.069*]
Online Purposes Count	0.026	0.016	1.668	0.096	[*-0.005*, 0.058]
Age	−0.352	0.201	−1.752	0.080	[*-0.747*, 0.043]
Gender	−0.075	0.066	−1.144	0.253	[*-0.205*, 0.054]
Education	0.251	0.065	3.885	<0.001*	[0.124, 0.378]
$R^2{=}0.141$, Adj. $R^2{=}0.133$, $F(5,572){=}18.73$, $p{<}0.001$					

*$p{<}0.05$. Age coded 1–5 (18–25 through 56+); Gender coded 1 = Male, 2 = Female; Education coded 1–5 (Primary through Other). Dependent variable: Ryff 18-item PWB composite total ($\alpha {=}0.727$)

## Discussion

Three principal findings from this cross-sectional study of post-COVID digital behavior and well-being warrant discussion.

First, the breadth of online activity—not merely screen time—is a meaningful correlate of adverse Health Complaints. The positive association between online purpose count and Health Complaints count ($\rho {=}0.202$) and the moderate effect size in the group comparison ($d{=}0.54$) suggest that individuals who engage in more diverse online activities report more Health Complaints. This aligns with research on “digital multitasking” and its cognitive and physiological costs [6, 18], and suggests that wellness interventions should target the diversity of simultaneous online activities, not only total screen time [41]. Recent experimental work reinforces this: short-term restriction of mobile internet access has been shown to improve sustained attention and well-being in controlled conditions [42], and structured “digital detox” protocols have produced measurable, if modest, improvements in affect and sleep [43–45]. Importantly, the partial Spearman correlation controlling for total screen time ($\rho _{\text {partial}}{=}0.154$, $p_{\text {perm}}{<}0.001$, 95% CI [0.07, 0.23]) confirms that activity diversity contributes to symptom burden independently of overall usage volume. However, the modest magnitude of this partial association indicates that breadth of online engagement explains only a small share of variance in Health Complaints; other unmeasured factors—such as content type, passive versus active use, or individual coping resources—likely play substantial roles. These results should therefore be interpreted as evidence of a statistically robust but practically small independent effect.

Second, within this sample of predominantly young urban adults, PWB was inversely associated with Health Complaints but not directly with online purpose count after FDR correction. The strongest bivariate association in the dataset was between PWB and Health Complaints ($\rho {=}{-}0.319$), consistent with literature linking Health Complaints to reduced well-being [19, 46]. The direct association between online purpose count and PWB was weak and non-significant after FDR correction. Descriptively (Analysis of indirect association patterns between online activity and well-being section), the product of the online-purposes–health-effects and health-effects–PWB associations was non-zero ($ab{=}{-}0.031$; 95% CI [*-0.044*, *-0.019*]), while the total bivariate association between online purposes and PWB was near zero. This decomposition is consistent with a statistical association pattern in which online activity breadth overlaps statistically with PWB primarily through its co-occurrence with self-reported Health Complaints, but the cross-sectional design does not permit inference about the direction of any underlying process [39]. Accordingly, these findings should be interpreted as statistical associations rather than evidence of underlying causal patterns. Prospective longitudinal and ecological-momentary-assessment designs are needed before a causal linkage can be tested [3, 8, 41]. The multivariate OLS regression (Multivariate predictors of psychological well-being section) is consistent with this descriptive pattern: Health Complaints count remained the strongest correlate of PWB after adjusting for age, gender, and education ($B{=}{-}0.091$, $p{<}0.001$), while online purpose count did not reach conventional significance ($p{=}0.096$). Education emerged as an independent positive correlate of PWB ($B{=}0.251$, $p{<}0.001$). We interpret education as a proxy for socioeconomic resources, including financial security, health literacy, and access to coping resources, which prior work links to eudaimonic well-being independently of behavioral factors; this warrants investigation in future studies [9, 46]. Recent large-scale cross-national work also cautions that population-level associations between digital technology adoption and well-being are often weaker and more heterogeneous than moral-panic narratives imply [15–17], underscoring the value of high-precision, sample-specific analyses such as the one presented here.

Third, within this demographic profile, the three-cluster solution reveals heterogeneity in digital behavior–health profiles. Rather than a uniform dose–response relationship, the data suggest distinct user segments. The “high Health Complaints, lower PWB” cluster ($n{=}189$) represents a sample-defined risk group warranting targeted intervention in replicated studies, while the “high multi-device” cluster ($n{=}291$) shows that intensive digital use does not necessarily coincide with poor health outcomes. These profiles may inform precision digital wellness approaches, pending replication in more demographically diverse samples. These clusters should be interpreted cautiously, as the relatively low silhouette scores indicate overlapping group boundaries; however, they provide meaningful exploratory insights into heterogeneous behavioral profiles.

The observed usage patterns across waves confirm a lasting pandemic digital footprint: post-2023 levels remain elevated relative to pre-COVID baselines across all three device types, indicating a structural shift in habits rather than a transient disruption [2, 3, 7]. Importantly, the observed usage differences across periods reflect between-wave comparisons rather than within-person longitudinal change; the same individuals were not tracked across time points.

### Limitations

Several limitations should be noted. First, the cross-sectional design precludes causal inference; observed associations may reflect reverse causation or confounding. Second, all measures are self-reported, introducing potential recall and social desirability biases [3, 41]. Third, retrospective self-report of pre-COVID and during-COVID device usage introduces substantial memory distortion. Participants were asked to recall usage from periods up to five years prior, rendering responses susceptible to telescoping bias (compressing temporally distant events into more recent memory) and mood-congruent recall (whereby current affect inflates retrospective estimates). Critically, the between-wave design does not track the same individuals across time periods; observed usage trajectories therefore reflect group-level estimates rather than true longitudinal change for the same respondents [3]. Future studies should employ prospective ecological momentary assessment (EMA) designs to capture real-time usage data across pandemic phases [8, 41]. Fourth, post-2023 usage variables had approximately 41% missingness, and the absence of a sensitivity analysis comparing imputed versus complete-case clustering results is a limitation. Fifth, participants were recruited through convenience sampling and were predominantly educated (87.5% undergraduate degree or above), urban (92.6%), single (89.1%), and aged 26–35 (98.8%). This demographic homogeneity substantially limits external validity. However, this population represents a group with high levels of digital engagement, making it particularly relevant for examining patterns of intensive digital behavior. Sixth, the internal consistency of the adapted Ryff scale was poor at the subscale level (all six subscales $\alpha {<}0.65$; range 0.228–0.624), attributable to short subscale length (2–4 items) and the adapted translation. This substantially limits subscale-level inferences; all PWB analyses were therefore restricted to the composite total score ($\alpha {=}0.727$). Future studies should use a fully validated Ryff translation with confirmed item-level psychometrics to support subscale-level comparisons [9, 36]. Additionally, the Health Complaints checklist has not been psychometrically validated; future studies should employ established instruments (PHQ-9, GAD-7, PSQI) for diagnostic precision [33–35]. Seventh, although multivariate regression was used to adjust for key demographic confounders (age, gender, and education), more advanced modelling approaches—such as hierarchical regression or longitudinal structural equation modelling applied to prospective data—would more precisely partition variance attributable to digital behaviour versus demographic factors and would permit formal testing of decomposition and moderation; this represents a primary direction for future research. Finally, clustering is exploratory and sensitive to feature selection and scaling choices. The demographic homogeneity of the sample — predominantly young (26–35), educated, and urban — and the between-wave rather than within-person design together constitute the primary constraints on generalizability; findings should be interpreted as descriptive evidence about a specific high-engagement population rather than the general adult population. In addition, the two waves used non-identical (subsequently harmonized) instrument versions, which constrains fine-grained cross-wave item comparability.

### Implications for practice and policy

Within this sample of predominantly young, educated, urban adults, the positive link between online purpose breadth and self-reported health symptom awareness points to a potentially modifiable risk factor. Digital wellness programs should consider addressing not just total screen time but the diversity and fragmentation of online activities [20, 41]. The sample-defined high-risk cluster (elevated Health Complaints, lower PWB, moderate usage) provides a candidate target profile for intervention studies combining digital hygiene guidance with physical activity promotion, given the established links between exercise and psychological well-being [37, 38].

From a public health perspective, these findings align with growing calls for digital health literacy programs and population-level mental health frameworks that account for post-pandemic technology use patterns [47, 48]. These descriptive findings may motivate future hypothesis-testing and intervention research on digital wellness assessment, particularly for young urban adults who represent the heaviest users of multi-purpose internet services [49, 50]. The cross-sectional association between online activity breadth, Health Complaints, and reduced PWB identified here provides a candidate entry point for preventive intervention research: reducing the adverse health symptom burden of intensive multi-purpose use may represent a more tractable lever for protecting well-being than attempting to reduce digital engagement wholesale [51–53]. These findings represent preliminary, descriptive evidence drawn from a convenience sample; replication with nationally representative samples would be required before policy recommendations could be made with confidence [42, 52, 54]. In the South Asian context specifically, related Bangladeshi evidence of elevated problematic internet use during the pandemic [21] suggests that locally adapted digital wellness programs could be particularly valuable. Implementation research further suggests that digital mental health supports are most useful when they are evidence-based, responsive to user context, and designed with attention to adoption, service quality, and real-world engagement patterns [55–60]. Related intervention and implementation research also points to the value of careful app design, usability testing, and evidence-based content when digital tools are deployed to support stress reduction and mental health in broader populations [61–66].

## Conclusion

In a convenience sample of 622 predominantly young, educated, urban adults, the breadth of online activity was significantly associated with self-reported Health Complaints, which in turn were inversely related to psychological well-being. Group-level estimates show higher mean usage during COVID relative to pre-COVID baselines, with a lower group-level estimate in Wave 2 (a between-wave, not within-person, comparison), consistent with prior studies on pandemic-related behavioral shifts [1, 3, 7]. These findings underscore the importance of addressing digital activity diversity—beyond screen time alone—in post-pandemic wellness strategies.

## Data Availability

The dataset generated and analyzed during the current study is publicly available in the Harvard Dataverse repository: https://doi.org/10.7910/DVN/W5GNLE. The dataset has been fully anonymized prior to publication.
